# Rotavirus Surveillance in Kisangani, the Democratic Republic of the Congo, Reveals a High Number of Unusual Genotypes and Gene Segments of Animal Origin in Non-Vaccinated Symptomatic Children

**DOI:** 10.1371/journal.pone.0100953

**Published:** 2014-06-26

**Authors:** Elisabeth Heylen, Bibi Batoko Likele, Mark Zeller, Stijn Stevens, Sarah De Coster, Nádia Conceição-Neto, Christel Van Geet, Jan Jacobs, Dauly Ngbonda, Marc Van Ranst, Jelle Matthijnssens

**Affiliations:** 1 Laboratory of Clinical and Epidemiological Virology, Department of Microbiology and Immunology, Rega Institute for Medical Research, University of Leuven, Leuven, Belgium; 2 Department of pediatrics, University Hospital Kisangani, Kisangani, the Democratic Republic of the Congo; 3 Department of pediatrics, University Hospital Leuven, Leuven, Belgium; 4 Department of Clinical Sciences, Institute of Tropical Medicine (ITM), Antwerp, Belgium; The University of Hong Kong, Hong Kong

## Abstract

Group A rotavirus (RVA) infections form a major public health problem, especially in low-income countries like the Democratic Republic of the Congo (COD). However, limited data on RVA diversity is available from sub-Saharan Africa in general and the COD in particular. Therefore, the first aim of this study was to determine the genetic diversity of 99 RVAs detected during 2007–2010 in Kisangani, COD. The predominant G-type was G1 (39%) and the most predominant P-type was P[6] (53%). A total of eight different G/P-combinations were found: G1P[8] (28%), G8P[6] (26%), G2P[4] (14%), G12P[6] (13%), G1P[6] (11%), G9P[8] (4%), G4P[6] (2%) and G8P[4] (1%). The second aim of this study was to gain insight into the diversity of P[6] RVA strains in the COD. Therefore, we selected five P[6] RVA strains in combination with the G1, G4, G8 (2x) or G12 genotype for complete genome analysis. Complete genome analysis showed that the genetic background of the G1P[6] and G12P[6] strains was entirely composed of genotype 1 (Wa-like), while the segments of the two G8P[6] strains were identified as genotype 2 (DS-1-like). Interestingly, all four strains possessed a NSP4 gene of animal origin. The analyzed G4P[6] RVA strain was found to possess the unusual G4-P[6]-I1-R1-C1-M1-A1-N1-T7-E1-H1 constellation. Although the majority of its genes (if not all), were presumably of porcine origin, this strain was able to cause gastro-enteritis in humans. The high prevalence of unusual RVA strains in the COD highlights the need for continued surveillance of RVA diversity in the COD. These results also underline the importance of complete genetic characterization of RVA strains and indicate that reassortments and interspecies transmission among human and animal RVAs strains occur regularly. Based on these data, RVA vaccines will be challenged with a wide variety of different RVA strain types in the COD.

## Introduction

Group A rotaviruses (RVAs) are the world’s leading cause of severe diarrhea in children <5 years of age and form a major public health problem, with an estimated 453,000 deaths per year worldwide. More than 50% of these deaths occur in Africa (232,000) and 7% of all RVA related deaths occur in the Democratic Republic of the Congo (COD) (32,653; 95% CI 27,804–37,699) [1]. In fact, the COD, together with India, Nigeria, Pakistan and Ethiopia, is one of the countries that bear the highest RVA mortality rates worldwide.

Despite this high burden of disease, only limited regional and country specific data on RVA genotypes diversity is available from sub-Saharan Africa in general and the COD in particular. The last report of the African Rotavirus Surveillance Network reported the distribution of predominant RVA genotypes among hospitalized children aged <5 years between 2007 and 2011 from 16 African countries [2]. The major G- and P-genotype combinations detected in this study were G1P[8] (18.4%), G9P[8] (11.7%), G2P[4] (8.6%), G2P[6] (6.2%), G1P[6] (4.9%), G3P[6] (4.3%), G8P[6] (3.8%) and G12P[8] (3.1%). Worldwide the majority of RVA strains infecting humans belong to the G1-G4, G9 and G12 G-genotypes, in combination with the P[4] (for G2) and P[8] (for G1, G3, G4, G9 and G12) genotypes. When focusing on the differences in the genetic setup of RVA strains circulating in Africa, compared to the ones circulating in industrialized countries, multiple studies have reported the high detection rate of RVA strains containing the P[6] and G8 genotypes. Both genotypes are believed to be of animal origin: P[6] RVA strains (most likely) originated from pigs, while G8 is a genotype often detected in cows or other members of the mammalian order of the *Artiodactyla* [3]–[10]. The majority of known human G8 RVAs strains have been described all over the African continent in combination with a large number of VP4 specificities and with different genetic backgrounds [11]–[23].

Two human RVA vaccines, Rotarix (GlaxoSmithKline) and RotaTeq (Merck) have been licensed and proven safe in large clinical trials in the Americas and Europe [24], [25]. Data from clinical trials conducted in five African countries have shown a lower vaccine immunogenicity and efficacy compared to the ones observed in America and Europe [26]–[28]. Despite this observed lower vaccine efficacy, vaccine introduction in Africa will prevent a greater number of RVA related deaths due to the substantially larger mortality rates in Africa compared to the Americas and Europe. Between August 2009 and May 2014, 21 African countries (in chronological order: South-Africa, Botswana, Morocco, Sudan, Ghana, Rwanda, Malawi, Tanzania, The Gambia, Burkina Faso, Ethiopia, Libya, Zambia, Burundi, Mali, Cameroon, Sierra Leone, The Republic of the Congo, Angola, Madagascar and Zimbabwe) have implemented RVA vaccination into their national immunization program [29], [30].

What factors determine the lower vaccine efficacy in Africa is not yet completely understood but both host (malnutrition, competing enteric pathogens, differences in histo-blood group antigens, the level of anti-rotavirus antibodies in breast milk and a lower immunological response) and viral (a higher RVA strain diversity) characteristics have been proposed to (partially) explain this observation [31]–[33].

Specific data on RVA strain diversity from the Democratic Republic of the Congo are scarce with only two currently available studies dealing with the RVA strain diversity in the COD. The first study analyzed the complete genomes of two G8 strains (RVA/Human-wt/COD/DRC86/2003/G8P[6] and RVA/Human-wt/COD/DRC88/2003/G8P[8]). These strains both possess a DS-1-like genotype constellation and are the only complete genomes of Congolese RVA strains available today [13]. The second study described the epidemiology of RVA associated gastroenteritis in Kinshasa, COD, from July to October in 2003, 2004, and 2005. The predominant genotypes in 2003 were G8P[6] and G8P[8] strains while in 2004 and 2005 G1P[6] strains were predominant [14].

The number of studies conducted in sub-Saharan Africa involving the analyses of complete genomes of human RVA strains isolated in Africa is also limited. To our knowledge the complete genotype constellation of 87 human RVA strains isolated in sub-Saharan Africa are retrievable from Genbank: 28 of the 87 RVA strains were isolated in Malawi, 22 in Cameroon, 20 in South-Africa, 4 in Kenya, 3 in Nigeria, 3 in Cote d’Ivoire, 3 in Zambia, 2 in the COD, 1 in Zimbabwe and 1 in Ethiopia [11], [13], [34]–[38]. Compared to the hundreds of complete genomes characterized from RVAs isolated in high-income countries, and considering the large number of co-circulating G/P genotype combinations, this number is rather low.

The present study aimed to determine the genetic diversity of RVAs detected during 2007–2010 from Kisangani, COD. In addition we selected five P[6] RVA strains in combination with the different observed G-genotypes (G1, G4, 2xG8 and G12) for complete genome analyses to gain insight into the diversity, origin and evolution of P[6] RVA strains in the COD.

## Methods

### Stool sample collection and detection of RVA

Fecal specimens were collected between May 2007 and December 2010 from children <5 years old admitted with gastroenteritis (GE) to the pediatric wards of three hospitals in Kisangani (University Hospital, General Referral Hospital Makisao of Kisangani – both public hospitals – and Village de Pédiatrie, a private hospital center) and screened by an immunochromatographic antigen test (Rota-CIT, BioConcept, Belgium) for the presence of RVA antigen. Fecal samples containing RVA were collected and shipped to the Rega Institute for Medical Research using chromatography paper strips as previously described [39].

### Rotavirus genotyping

The paper strips were inserted into an Eppendorf tube with 500 µl of universal transport medium (Copan Diagnostics, Corona, CA, USA) and thoroughly squeezed using sterile forceps. An aliquot of 140 µl of the squeezed eluate was used for RNA extraction using the QIAamp Viral RNA mini kit (QIAGEN/Westburg, Leusden, The Netherlands) according to the manufacturer’s instructions. The G- and P-genotypes of 99 antigen-positive samples were characterized by reverse-transcription polymerase chain reaction (RT-PCR) using the QIAGEN OneStep RT-PCR kit (QIAGEN/Westburg) using primers Beg9 and End9 (VP7), and VP4_1-17F and Con2 (VP4). Primer sequences are shown in table S1. PCR products were run on a polyacrylamide gel, stained with EtBr and visualized under UV-light. The positive samples were purified with ExoSAP-IT (Affymetrix, Santa Clara, CA, USA), and Sanger sequenced using the forward primers (Beg9 or VP4_1-17F) with the ABI PRISM BigDye Terminator cycle sequencing reaction kit (Applied Biosystems, Foster City, CA, USA).

### Sequencing complete genomes

For the complete genomes, RT-PCR was carried out with an initial reverse transcription step at 50°C for 30 min followed by a PCR activation step at 95°C for 15 min, 35 cycles of amplification, and a final extension step for 10 min at 70°C in a Biometra T3000 thermocycler (Biometra, Westburg BV, Netherlands). For the gene segments encoding VP6, VP7, NSP1, NSP2, NSP3, NSP4 and NSP5, the amplification cycle conditions were as follows: 30 s at 94°C, 30 s at 45°C, and 2 min at 72°C. For the larger segments encoding VP1, VP2, VP3 and VP4 the cycle conditions were 30 s at 94°C, 30 s at 48°C, and 6 min at 72°C. Primers used to amplify the VP1, VP2, VP3, VP6, NSP1, NSP2, NSP3, NSP4 and NSP5 gene segments are described in table S1. Primer walking was used for full-length sequencing of the longest genes (VP1–VP4). The 5′ and 3′ terminal sequences of the 11 genomic segments were determined for all five strains using a modified RACE technique as previously described [40].

### Sequence alignment and phylogenetic analysis

The Mega 5.10 software was used for phylogenetic and molecular evolutionary analyses [41]. To compute evolutionary distances we used the p-distance nucleotide substitution model. In phylogenetic analyses we opted for maximum likelihood phylogenetic analyses using the complete deletion function in order to include partially sequenced genes. Bootstrap resampling analysis (500 replicates) was performed to measure the reliability of the tree topologies.

### Nucleotide sequence accession numbers

Accession numbers from sequences of this study: KJ870690-KJ870932.

### Ethics Statement

Pending the installation of an ethical committee in the Oriental Province of the COD and at the Kisangani hospitals, the study was assessed and approved by the Provincial Health Officer (the highest ranked medical authority in the Oriental Province) and the Director’s Board of UH Kisangani, General Reference Hospital of Makiso and Village de Pédiatrie, respectively. All samples were collected in a standard of care setting for therapeutic or diagnostic reasons. There has been no influence on the treatment of the patients based on the results of the RVA detection test, and analyses for this study were performed after installment of the patients’ treatment. Data were fully anonymized at collection.

## Results

### 1. Rotavirus incidence from May 2007 to December 2010

#### RVA detection rate and study data

**Figure 1:**
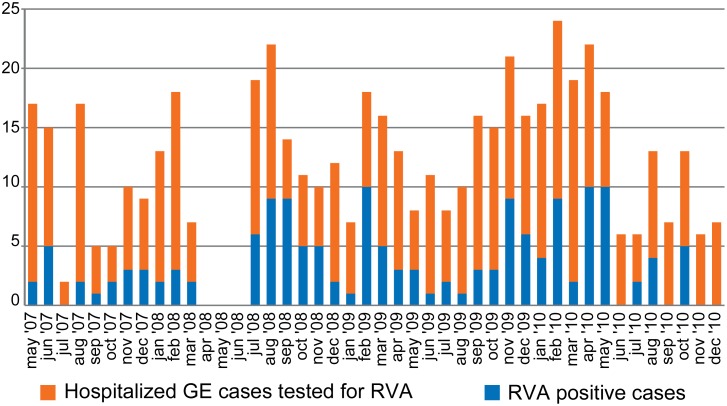
Numbers of children hospitalized with GE (blue bars) and children hospitalized with GE tested RVA positive (red bars) per month from May 2007 until December 2010.

A total of 524 fecal specimens were collected over the entire study period of which 154 samples (29%) tested positive with the immunochromatographic antigen test. Figure 1 shows the number of children hospitalized with gastroenteritis (GE) and the number of RVA positive cases over the entire study period. There were clear fluctuations in the number of RVA positive cases and in the number of hospitalized GE cases per month with a three-month long period (April–June 2008) during which no samples were collected because of stock rupture. Duration of hospitalizations associated with RVA varied between 1 and 10 days and occurred among children aged 1 week-2 years, with a mean age of 10 months.

**Figure 2:**
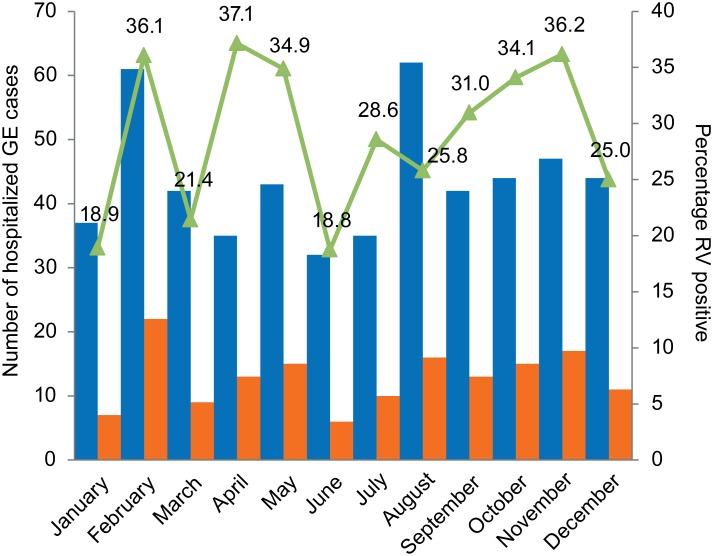
Number of hospitalized GE cases (blue bars) and number of hospitalized GE cases tested RVA positive (red bars) pooled per month over the entire study period. The green line indicates the percentage RVA positive cases tested in each month.

To determine a possible seasonal variability in RVA disease in Kisangani, all data shown in figure 1 were pooled per month and plotted together with the percentage of RVA positive cases in figure 2. The proportion of RVA positive cases among children hospitalized with GE ranged between 18.8% and 37.1%, with an average of 29.0%. RVA infections occurred year-round with three periods of increased RVA detection rates: February, April–May and September–November.

### 2. Genotype distribution

#### G- and P- genotyping of rotavirus strains

**Figure 3:**
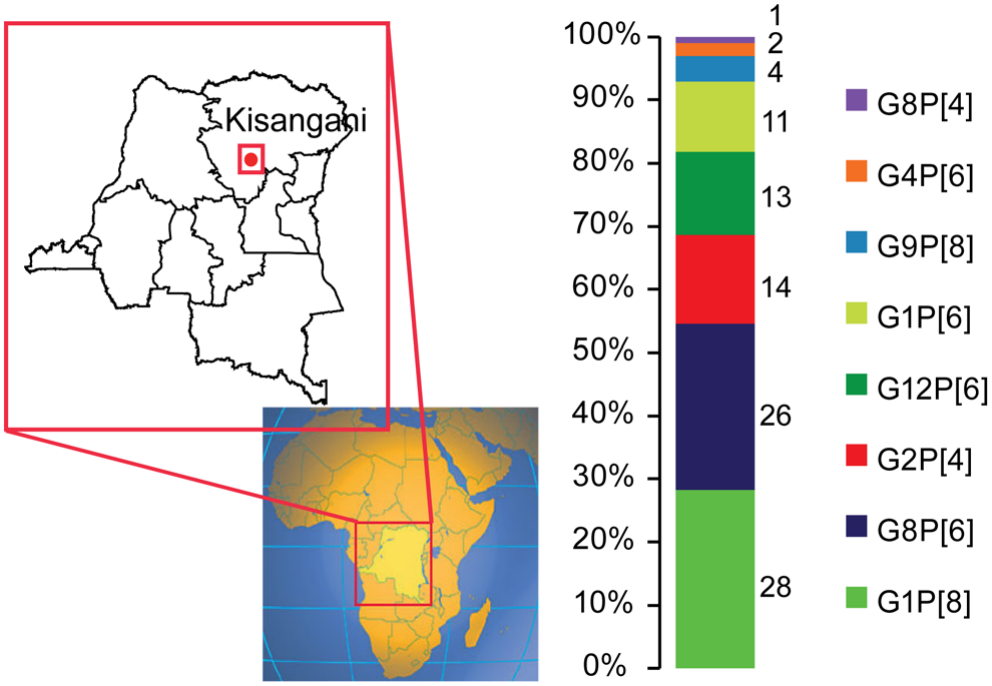
Geographic location of the Democratic Republic of the Congo on the African continent; location of the city of Kisangani in the COD together with the genotype distribution of the 99 analyzed RVA strains. Percentages of the different genotypes are indicated on the left. The number of strains per genotype is shown on the right hand side.

Ninety-nine samples out of 154 samples were selected, based on the availability of sufficient stool, for further G and P-genotyping. In the analyzed samples the predominant G-type was G1 (detected in 39% of specimens) and the most predominant P-type was P[6] (53%). A total of eight different G/P-combinations were found: G1P[8] (N = 28), G8P[6] (N = 26), G2P[4] (N = 14), G12P[6] (N = 13), G1P[6] (N = 11), G9P[8] (N = 4), G4P[6] (N = 2) and G8P[4] (N = 1) (figure 3). Only 46% of the isolates were found with the most typical human RVA genotype combinations (G1P[8], G2P[4] and G9P[8]). In contrast, 53% of the strains were found with the P[6] genotype, in combination with the VP7 genotypes G8, G1, G12 or G4.

**Figure 4:**
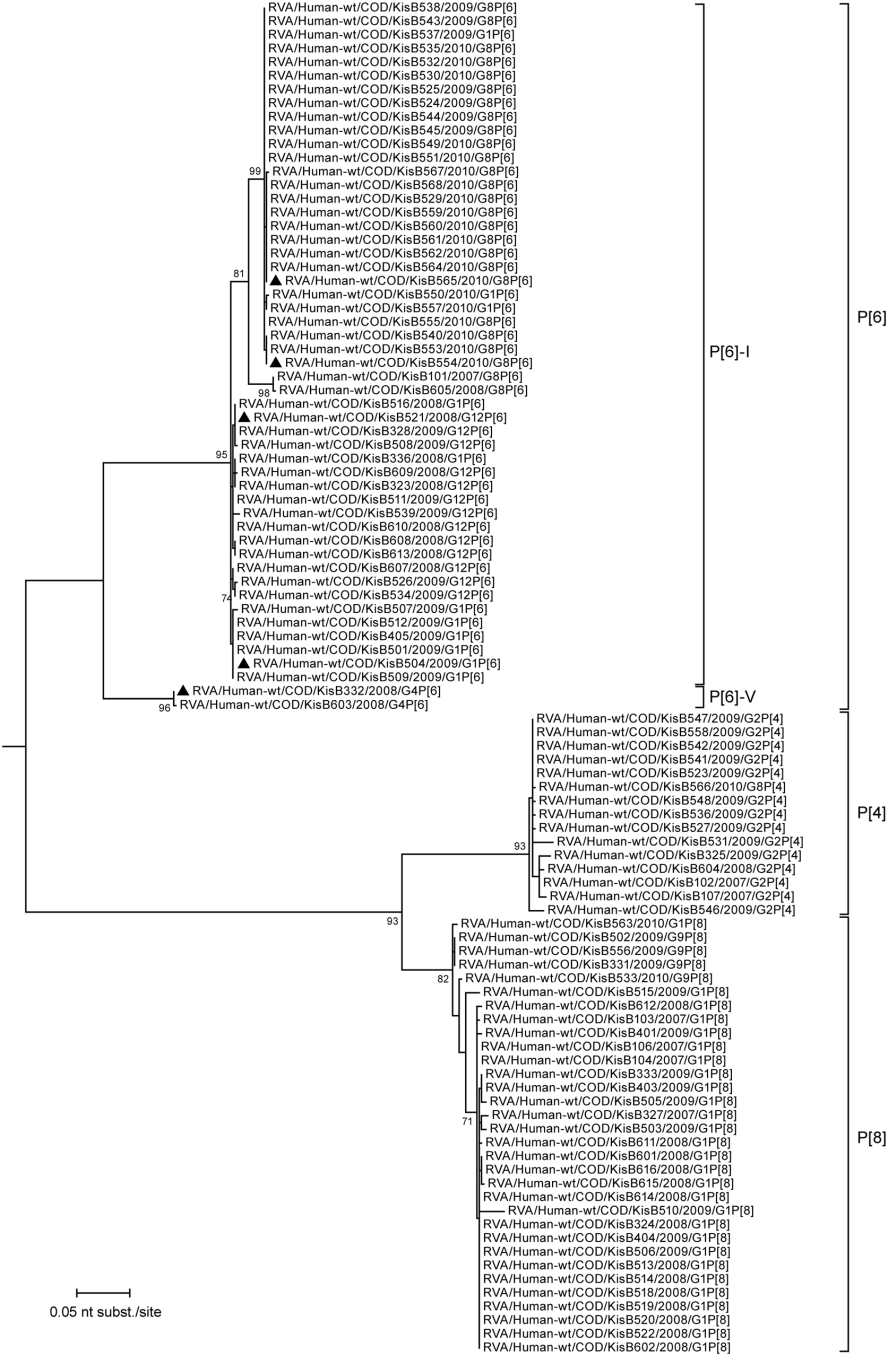
Maximum likelihood tree based on 496 nucleotides of the VP4 encoding gene of the 99 RVA strains characterized in this study. Tree was rooted using RVA/Human-tc/JPN/AU-1/1982/G3P[9] as an outgroup. Bootstrap values (500 replicates) above 70 are shown. The strains completely analyzed in this study are marked with a black triangle.

**Figure 5:**
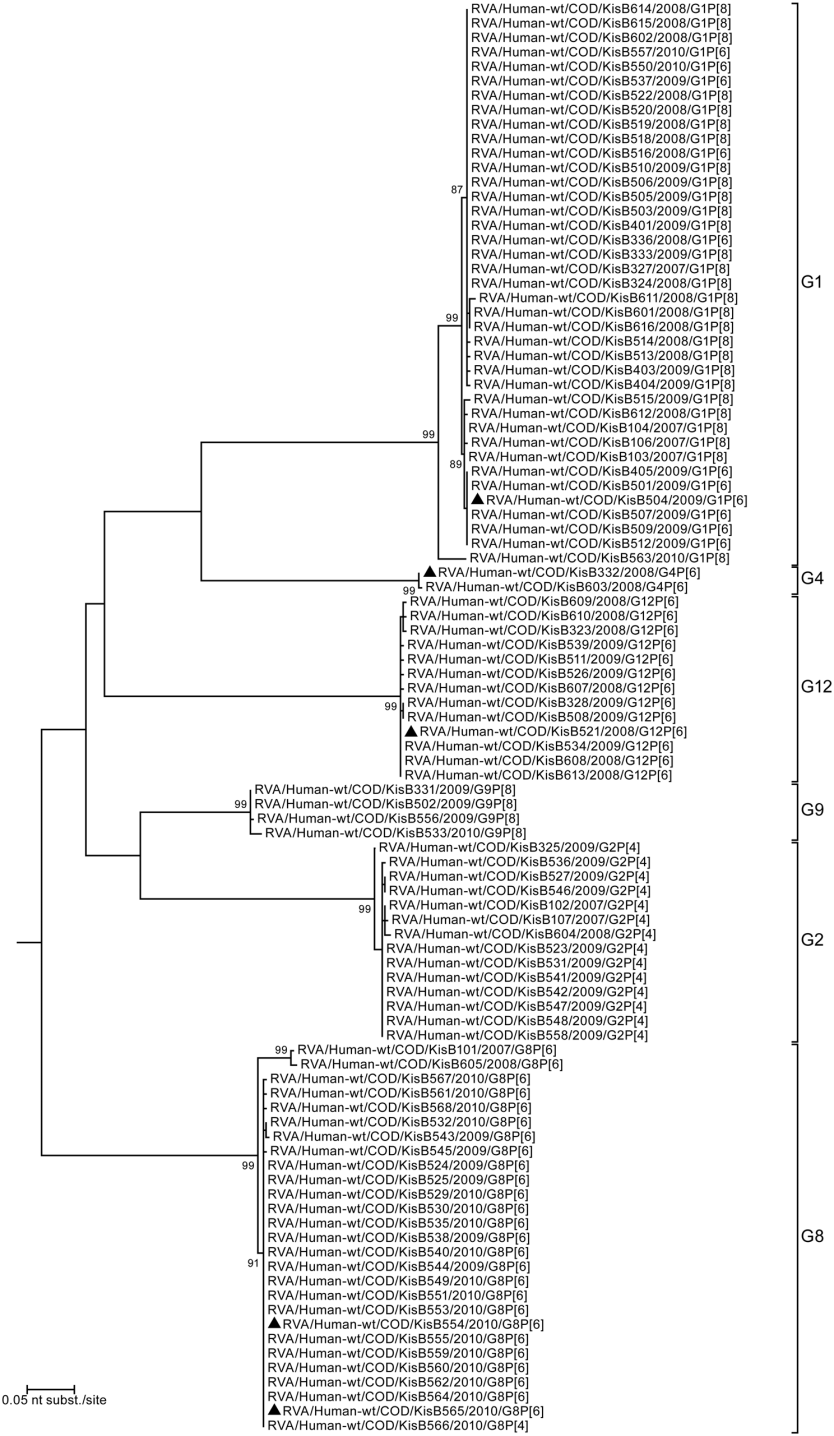
Maximum likelihood tree based on 363 nucleotides sequence of the VP7 encoding gene of the 99 RVA strains characterized in this study. Tree was rooted using RVA/Pigeon-tc/JPN/PO-13/1983/G18P[17] as an outgroup. Bootstrap values (500 replicates) above 70 are shown. The strains completely analyzed in this study are marked with a black triangle.

To investigate the genetic diversity within the VP4 and VP7 genes isolated in this study phylogenetic trees based on the partial nucleotide sequences of both genes were constructed (figures 4–5). For VP4, the majority (53%) of the characterized RVA strains clustered within the P[6] genotype, more specifically in two previously defined lineages, P[6]-I and P[6]-V, both of them containing human and porcine strains [10]. The Congolese RVA strains belonging to P[6]-I lineage analyzed in this study can be divided in three different clusters, two large and one smaller cluster (figure 4). The first large cluster contains the majority (24/26) of G8P[6] strains while the second big cluster contains all G12P[6] strains. G1P[6] strains were found in both the G8P[6] as the G12P[6] clusters (figure 4). The third, smaller cluster contains only 2 G8P[6] strains: RVA/Human-wt/COD/KisB101/2007/G8P[6] and RVA/Human-wt/COD/KisB605/2008/G8P[6], the only two G8P[6] strains isolated before 2009. Both G4P[6] strains detected in this study (KisB332 and KisB503) belonged to the P[6]-V lineage, a lineage containing mostly porcine or human strains believed to be of (partial) porcine RVA origin. Both samples were isolated in 2008, only one month apart from each other in the same hospital and shared 99.7% nucleotide (nt) identity in their VP4 genes.

All P[4] strains were found in combination with the G2 genotype, except for a single G8 strain (KisB566). All G2P[4] strains were very closely related to each other despite the fact that they were isolated over the entire study period. The fact that the VP4 gene of KisB566 was almost identical to the G2P[4] strains suggests a rather recent reassortment event involving the VP7 gene. The P[8] strains from Kisangani showed limited diversity, with almost identical G1P[8] strains circulating in the Kisangani area between 2007 and 2009. The only characterized G1P[8] strain isolated in 2010 (RVA/Human-wt/COD/KisB563/2010/G1P[8]) was found to cluster closer to the four detected G9P[8] strains than to the other G1P[8] strains characterized in this study, again suggesting a recent reassortment event.

For VP7, we found limited diversity within each G-genotype, especially within G2, G4, G9 and G12 were we found at least 97.9%, 99.2%, 98.7% and 98.2% of nt similarity, respectively. Within the G1 genotype, two clusters could be defined, one of which contains only a single Kisangani strain: RVA/Human-wt/COD/KisB563/2010/G1P[8] (figure 5). This strain is the only G1P[8] RVA strain isolated in 2010, and shows 94.7% –95.7% nt similarity with G1P[8] strains isolated between 2007–2009. The large G1 cluster showed strains with P[6] and P[8] intermingled, further indicating the frequent occurrence of reassortments. A clustering according to year of isolation can also be found within the G8 RVA strains. More specifically, strains isolated in 2007 or 2008 and strains isolated in 2009 or 2010 differ 3.6% to 4.7% on the nucleotide level.

Both for VP4 and VP7, all strains isolated in Kisangani were closely related to RVAs present in the global RVA data collection, in particular to strains isolated from other African patients. For more detail, extended trees for both VP4 and VP7 were added as supporting information (Figure S1 for VP4 and Figure S2 for VP7). Both trees contain twelve representative strains from this study together with RVA VP4 or VP7 sequences available in Genbank.

### 3. Complete genome analysis

#### Genotype constellations of whole genomes

To gain insight into the diversity, origin and evolution of P[6] RVA strains in the COD we selected P[6] strains, based on the phylogenetic clustering in the VP7 tree and on the viral load of the samples. In total, the nucleotide sequences of the eleven genes of five P[6] RVA strains with the different G-genotypes detected in this study (G1, G4, 2xG8 and G12) were completely determined, except from the first 33 nucleotides of the VP2 gene of strain KisB332 which could not be completed due to the lack of sample available to complete the analysis. Complete genotype assignments of the five fully sequenced P[6] strains together with a number of other representative strains are shown in table 1.

**Table 1:** RVA genotype constellations of the P[6] strains characterized in the present study (in bold) together with selected human and porcine P[6] RVA strains.

	VP7	VP4	VP6			VP1	VP2	VP3	NSP1	NSP2	NSP3	NSP4	NSP5
G1P[6] strains													
** RVA/Human-wt/COD/KisB504/2009/G1P** [6]	**G1**	**P** [6]			**I1**	**R1**	**C1**	**M1**	**A1**	**N1**	**T1**	**E1**	**H1**
RVA/Human-wt/BRA/BA17290/2009/G1P[6]	G1	P[6]			*I1*	*R1*	*C1*	*M1*	A1	N1	T1	E1	H1
RVA/Human-tc/JPN/AU19/1997/G1P[6]	G1	*P* [*6*]			*I5*	*R1*	*C1*	*M1*	*A8*	*N1*	*T1*	*E1*	H2
G12P[6] strains													
** RVA/Human-wt/COD/KisB521/2008/G12P** [6]	**G12**	**P** [6]		**I1**		**R1**	**C1**	**M1**	**A1**	**N1**	**T1**	**E1**	**H1**
RVA/Human-wt/ZAF/3176WC/2009/G12P[6]	G12	P[6]		I1		R1	C1	M1	A1	N1	T1	E1	H1
RVA/Human-wt/ZMB/MRC-DPRU3491/2009/G12P[6]	G12	P[6]		I1		R1	C1	M1	A1	N1	T1	E1	H1
RVA/Human-wt/BGD/Dhaka12/2003/G12P[6]	G12	P[6]		I1		R1	C1	M1	A1	N1	T1	E1	H1
RVA/Human-wt/DEU/GER172/2008/G12P[6]	G12	P[6]		I1		R1	C1	M1	A1	N1	T1	E1	H1
G4P[6] strains													
** RVA/Human-wt/COD/KisB332/2008/G4P** [6]	**G4**	**P** [6]		**I1**		**R1**	***C1***	**M1**	**A1**	**N1**	**T7**	**E1**	**H1**
Hungarian strains BP190, BP1792 and BP1490	G4	P[6]		I1		*R1*	C1	M1	A1	*N1*	*T7*	E1	H1
Hungarian strains BP271, BP1125 and BP1547	G4	P[6]		I1		*R1*	C1	M1	A8	*N1*	T7	E1	H1
Hungarian strains BP1227 and BP1231	G4	P[6]		I1		*R1*	C1	*M1*	A1	*N1*	T1	E1	H1
RVA/Human-tc/GBR/ST3/1975/G4P[6]	G4	P[6]		I1		R1	C1	M1	A1	N1	T1	E1	H1
RVA/Pig-tc/USA/Gottfried/1983/G4P[6]	G4	P[6]		I1		R1	C1	M1	A8	N1	T1	E1	H1
RVA/Human-wt/ARG/Arg4671/2006/G4P[6]	G4	P[6]		*I1*		*R1*	*C1*	*M1*	*A8*	N1	*T1*	E1	*H1*
RVA/Human-wt/ARG/Arg4605/2006/G4P[6]	*G4*	*P* [*6*]		*I1*		*R1*	*C1*	M1	*A8*	N1	*T7*	E1	*H1*
RVA/Human-wt/BRA/HSP180/1999/G4P[6]	*G4*	*P* [*6*]		*I1*		*R1*	*C1*	*M1*	*A8*	N1	*T7*	E1	*H1*
RVA/Human-wt/BRA/NB150/1997/G1G4P[6]	*G1G4*	P[6]		*I1*		*R1*	*C1*	*M1*	A1	Nx	*T1*	E1	*H1*
RVA/Human-tc/CHN/R479/2004/G4P[6]	G4	P[6]		I5		R1	C1	M1	A1	N1	T7	E1	H1
RVA/Human-wt/CHN/GX54/2010/G4P[6]	G4	P[6]		I1		R1	C1	M1	A8	N1	T1	E1	H1
RVA/Human-wt/IND/mani-362/2007/G4P[6]	G4	P[6]		I1		*R1*	*C1*	*M1*	A8	N1	T1	E1	H1
RVA/Human-wt/BRA/HST327/1999/G4P[6]	G4	P[6]		Ix		*R1*	*C1*	*M1*	*A1*	N1	*T1*	E1	*H1*
G8P[6] strains													
** RVA/Human-wt/COD/KisB554/2010/G8P** [6]	**G8**	**P** [6]		**I2**		**R2**	**C2**	**M2**	**A2**	**N2**	**T2**	**E2**	**H2**
** RVA/Human-wt/COD/KisB565/2010/G8P** [6]	**G8**	**P** [6]		**I2**		**R2**	**C2**	**M2**	**A2**	**N2**	**T2**	**E2**	**H2**
RVA/Human-wt/COD/DRC86/2003/G8P[6]	G8	P[6]		I2		R2	C2	M2	A2	N2	T2	E2	H2
RVA/Human-tc/MWI/QEC29/2005/G8P[6]	*G8*	*P* [*6*]		*I2*		*R2*	*C2*	*M2*	*A2*	*N2*	*T2*	E2	*H2*
RVA/Human-tc/MWI/MW1-860/1999/G8P[6]	*G8*	*P* [*6*]		*I2*		*R2*	*C2*	*M2*	*A2*	*N1*	*T2*	E2	*H1*
RVA/Human-wt/CMR/6809/2000/G8P[6]	*G8*	*P* [*6*]		*I2*		*R1*	*C1*	*M1*	*A1*	*N1*	*T1*	*E1*	*H1*
RVA/Human-wt/CMR/6782/2000/G8P[6]	*G8*	*P* [*6*]		*I1*		*R1*	*C1*	*M1*	*A1*	*N1*	*T1*	*E1*	*H1*

Gene segments of which no complete open reading frame was available in Genbank are indicated in italic.

The genetic background of the G1P[6] and G12P[6] strains was entirely composed of genotype 1 (Wa-like). To our knowledge, no strain with the same genotype constellation as RVA/Human-wt/COD/KisB504/2009/G1P[6] has previously been reported from Africa. However, the same constellation was reported in a study investigating 6 Brazilian G1P[6] RVA strains, isolated from vaccinated children in 2009 and 2010 [42]. The G12-P[6]-I1-R1-C1-M1-A1-N1-T1-E1-H1 configuration has been previously reported in two African countries: South-Africa (RVA/Human-wt/ZAF/3176WC/2009/G12P[6]) and Zambia (RVA/Human-wt/ZMB/MRC-DPRU3491/2009/G12P[6]) [35]. Both G8P[6] strains possessed a conserved genomic configuration: G8-P[6]-I2-R2-C2-M2-A2-N2-T2-E2-H2. This constellation was exactly the same as that of RVA/Human-wt/COD/DRC86/2003/G8P[6], also isolated in the COD, and RVA/Human-tc/MWI/QEC29/2005/G8P[6], isolated in Malawi [11], [13].

The G4P[6] strain was found to possess the, for humans unusual, G4-P[6]-I1-R1-C1-M1-A1-N1-T7-E1-H1 genotype constellation. Although unusual in humans, a recent study describing G4P[6] strains in Hungarian children, reported three strains with the exact same genotype constellation as KisB332 [43]. The genotype constellation I1/I5-R1-C1-M1-A1/A8-N1-T1/T7-E1-H1 is typically found in pigs with a variety of different G- and P-genotypes [9], [44]–[46]. A number of recent studies have identified human G4P[6] strains detected in different parts of the world, which are believed to be examples of direct interspecies transmissions, or may have undergone reassortments with human RVA strains [43], [47]–[51].

#### Phylogenetic analyses

**Figure 6:**
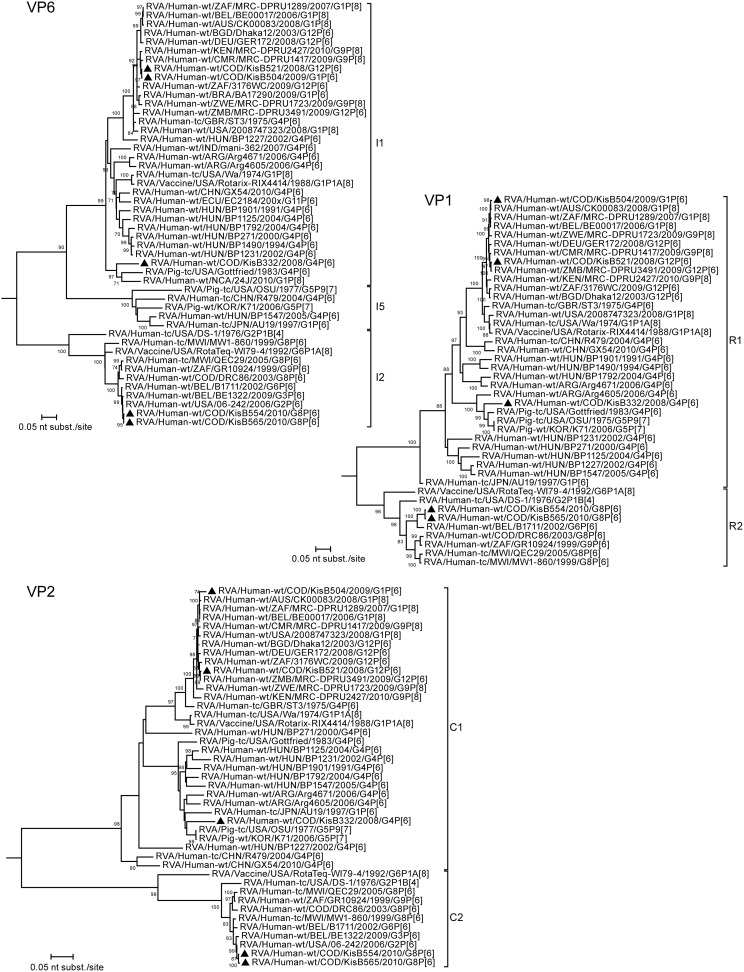
Maximum likelihood trees inferred from the nucleotide sequences of RVA VP6, VP1 and VP2 genes. The gene sequence lengths compared were: VP6 (1064 bp), VP1 (3168 bp), VP2 (2534 bp). The bootstrap values (500 replicates) are shown at the branch nodes (values <70% not shown). The scale bar is proportional to the genetic distance. An outgroup sequence (RVA/Pigeon-tc/JPN/PO-13/1983/G18P[17]) for each gene was included. Completely sequenced RVA strains from Kisangani are indicated with black triangles.

**Figure 7:**
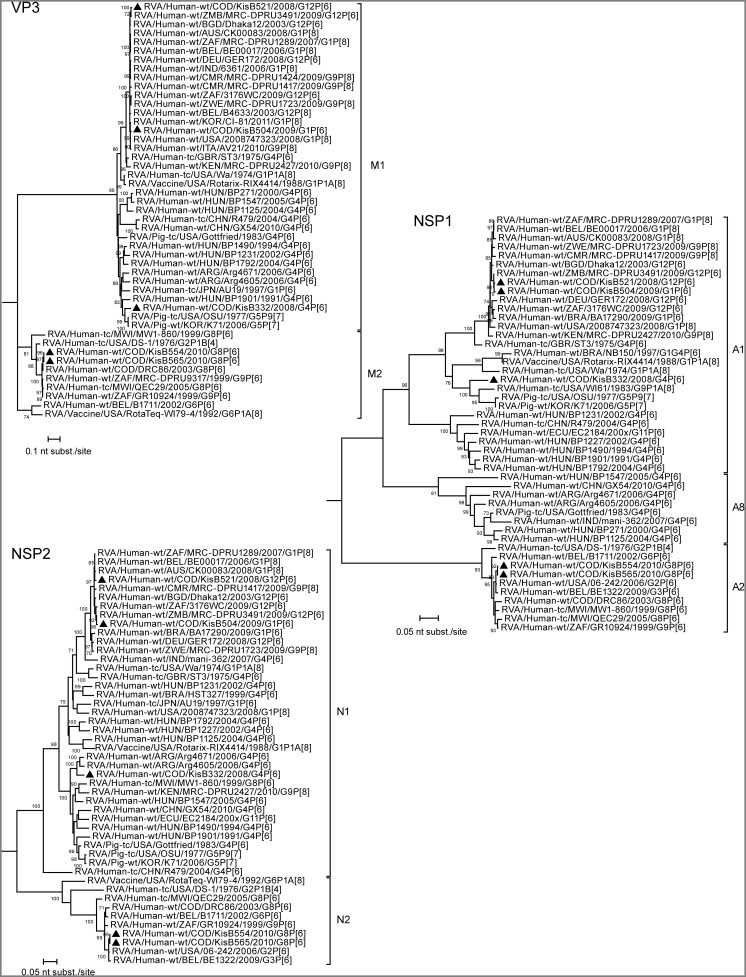
Maximum likelihood trees inferred from the nucleotide sequences of RVA VP3, NSP1 and NSP2 genes. The gene sequence lengths compared were: VP3 (2442 bp), NSP1 (1139 bp), NSP2 (938 bp). The bootstrap values (500 replicates) are shown at the branch nodes (values <70% not shown). The scale bar is proportional to the genetic distance. An outgroup sequence (RVA/Pigeon-tc/JPN/PO-13/1983/G18P[17] for VP3 and NSP2 and RVA/Horse-wt/ARG/E403/2006/G14P[12] for NSP1) was included. Completely sequenced RVA strains from Kisangani are indicated with black triangles.

**Figure 8:**
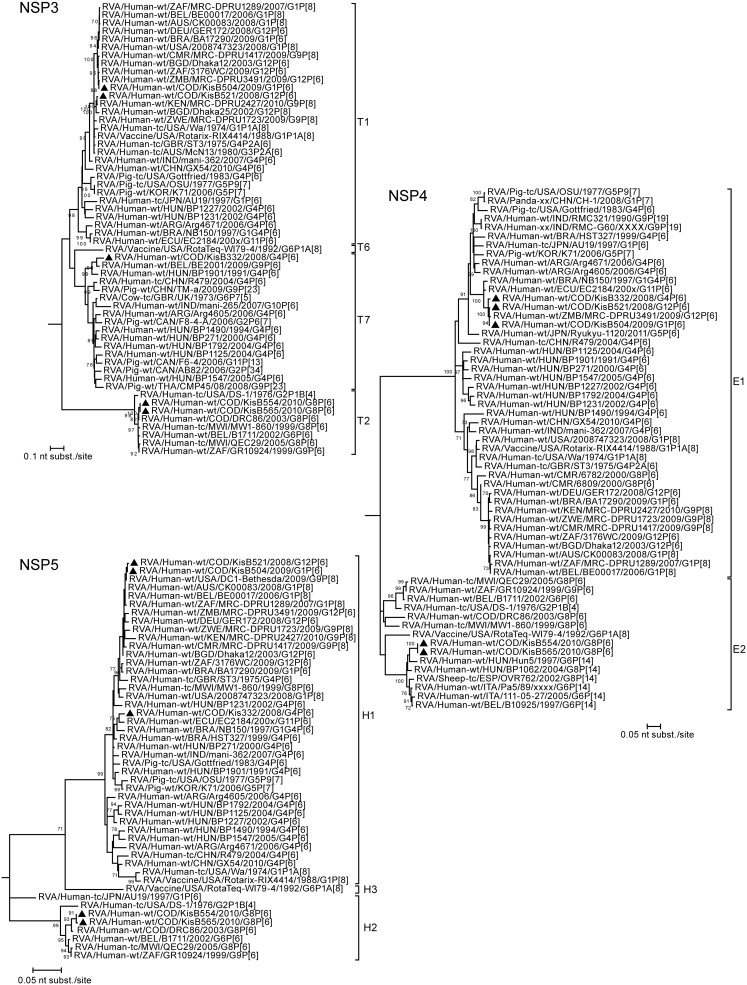
Maximum likelihood trees inferred from the nucleotide sequences of RVA NSP3, NSP4 and NSP5 genes. The gene sequence lengths compared were: NSP3 (841 bp), NSP4 (501 bp), NSP5 (542 bp). The bootstrap values (500 replicates) are shown at the branch nodes (values <70% not shown). The scale bar is proportional to the genetic distance. An outgroup sequence (RVA/Pigeon-tc/JPN/PO-13/1983/G18P[17]) for each gene was included. Completely sequenced RVA strains from Kisangani are indicated with black triangles.

Despite the different G-genotype, a very close genetic relationship exists between the G1P[6] (KisB504) and G12P[6] (KisB521) strain within their segments VP4 (99.6%), VP6 (99.9%), NSP1 (99.6%), NSP4 (99.8%) and NSP5 (99.8%). For the remaining genome segments encoding VP1, VP2, VP3, NSP2 and NSP3 a slightly lower nucleotide sequence similarity was detected, ranging from 94.4% (for NSP3) to 98.2% (for NSP2). The genetic similarity between the VP1-4, VP6, VP7, NSP1-3 and NSP5 of RVA strains KisB521, KisB504 and other recently isolated human Wa-like RVA strains from all over the world was very high (figure 4–8). The majority of gene segments of strain KisB521 (VP7, VP4, VP1-3, NSP1, NSP4) and five gene segments of strain KisB504 (VP4 and NSP1-4) are closely related to a recently isolated strain from Zambia (RVA/Human-wt/ZMB/MRC-DPRU3491/2009/G12P[6]).

Both G8P[6] strains clustered very closely together in the phylogenetic trees of all gene segments, with a nucleotide sequence similarity ranging between 99.5% and 100% (figure 4–8). These results indicate a clonal origin of both strains, which were isolated within a 2-month interval, from patients admitted to the same hospital. For VP7, VP3, NSP3 and NSP5 these strains shared the highest nucleotide similarity with RVA/Human-wt/COD/DRC86/2003/G8P[6]. Human RVA strain DRC86 is the only completely characterized Congolese G8P[6] strain available in Genbank until now [13]. Maximal nucleotide sequence identity of KisB554 and KisB565 was found with RVA strain RVA/Human-wt/USA/06-242/2006/G2P[6] (VP4, VP6, VP2, NSP1 and NSP2) and RVA/Human-wt/BEL/B1711/2002/G6P[6] (VP1). Strain 06–242 was isolated in an American child in 2006 and was the first G2P[6] strain characterized with a DS-1-like genotype constellation [52]. Strain B1711 also possessed a complete DS-1-like genotype constellation and was isolated from a 13-month-old child admitted to the university hospital in Leuven, Belgium, with severe gastroenteritis after returning from a trip to Mali [53], [54].

Overall, most gene segments of the G8P[6], G1P[6] and G12P[6] strains appeared to cluster closely to gene segments of recently isolated human RVA strains from all around the world, with the exception of NSP4. This gene segment showed more similarity with porcine/human porcine-like (for the G1P[6], G12P[6] and G4P[6] strains) or bovine/human bovine-like (for the G8P[6] strains) RVAs (figure 8). More specific, the three Congolese strains (and the Zimbabwean strain RVA/Human-wt/ZMB/MRC-DPRU3491/2009/G12P[6]) belonging to genotype E1 clustered together with RVA strains RVA/Human-wt/BRA/NB150/1997/G1G4P[6] and RVA/Human-wt/ECU/EC2184/200x/G11P[6], both previously classified as porcine-human reassortants [51], [55]. Furthermore, these six strains also felt into a larger cluster containing porcine RVA strains, or strains believed to be of porcine origin. On the other hand, the NSP4 encoding gene segments of the G8P[6] strains belonged to an E2 genotype cluster containing both animal and human P[14] RVA strains, which are believed to be the result of direct interspecies transmissions from sheep or other members of the mammalian order *Artiodactyla* [6].

For the G4P[6] strain (KisB332) it appeared that all gene segments were more closely related to porcine RVA strains or human porcine-like RVA strains than to typical human RVA strains. A possible exception is VP7, because it is difficult to determine the exact origin of the VP7 gene, since there are no porcine G4 RVA sequences from Africa known today. In general, the gene segments of KisB332 showed lower nucleotide sequence identities to known contemporary and historic human or animal RVAs, especially in the VP6, VP1, VP2 and NSP1 genes (figures 6 and [7]). Although these gene segments of KisB332 were only relatively distantly related to known RVA strains, they showed the highest relatedness to RVA/Human-wt/NCA/24J/2010/G1P[8] (for VP6), RVA/Pig-tc/USA/OSU/1977/G5P9[7] (VP1), RVA/Pig-wt/KOR/K71/2006/G5P7 (VP2) and human reference strain RVA/Human-tc/USA/WI61/1983/G9P1A[8] (NSP1) [56], [57]. The VP3 of KisB332 was most closely related to porcine RVA strain OSU (figure 7). The VP4, NSP2, NSP3, NSP4 and NSP5 genes clustered together with putative porcine interspecies transmitted RVA strains or human-porcine reassortant strains (BE2001, Arg4671, NB150, EC2184) [47], [51], [55], [58]. Interestingly, the NSP3 gene segment of KisB332 belongs to the rarely described T7 genotype (figure 8). The T7 genotype was first detected in a bovine RVA strain (RVA/Cow-tc/GBR/UK/1973/G6P7[5]) and was more recently described in a bovine-like human strain (RVA/Human-xx/IND/Mani-265/2007/G10P[6]) [48],[59]. However, an increasing number of studies have reported the T7 genotype in porcine-like human strains (R479, BE2001, BP1901, BP1490, BP271, BP1792, BP1125, BP1547 and Arg4605) and porcine RVA strains (CMP45, AB82, F6–4, F8–4-A and TM-a) derived from different geographical locations [43], [46], [47], [50], [58]. The detection of the T7 genotype in strain RVA/Human-wt/COD/KisB332/2008/G4P[6], a strain with a human porcine-like gene constellation, makes it more likely that T7 genotypes have their origins in pigs as was previously suggested in other studies [46], [58].

## Discussion

Data shown in figure 2, suggest three periods of increased RVA detection rates over the course of this study. However, these fluctuations can be partially explained by sampling bias inherent to the circumstances in which these samples have been collected. Kisangani is known for its complex political and economical climate, making it difficult to collect sufficient samples over a large period of time without any period of lower or no sampling. Although the data available in this study do have their limitations, the available data confirm previous studies that reported year-round RVA infections in tropical settings, with ups and downs but no clear period with minimal or no RVA hospitalizations [60], [61].

The six most common G/P-genotype combinations in countries with a more moderate climate and by extension worldwide are G1P[8], G2P[4], G3P[8], G4P[8], G9P[8] and G12P[8]. In this study a high proportion of P[6] RVA strains was observed together with a large number of unusual strains (G8P[6], G12P[6], G1P[6], G4P[6] and G8P[4]). These unusual genotype combinations may represent potential reassortants between human and animal RVA strains as a result of zoonotic infection. The only way to investigate this is to perform phylogenetic analysis of complete genomes. Because of the high number of P[6] RVA strains detected in this study and the fact that the P[6] genotype is much less prevalent in high-income countries this study focused on the four different P[6] genotype combinations detected in Kisangani between May 2007 and December 2010. The majority of the gene segments from the five P[6] strains characterized in this study appeared to have a genetic background most closely related to human RVA strains circulating all over the world. However, all five strains possessed at least one gene segment (NSP4) that was most closely related to animal RVAs. One strain, RVA/Human-wt/COD/KisB332/2008/G4P[6], possessed a majority of gene segments (if not all) most closely related to porcine, or human porcine-like RVA strains. For most genome segments KisB332 had still a considerable genetic distance to known porcine RVA strains. This can partly be explained by sampling bias since surveillance studies for animal rotaviruses are much less common than human rotavirus surveillance studies. In addition, porcine RVA sequences obtained from African pigs are not available in Genbank thus far. The lack of availability of complete genome sequences from porcine RVA strains in general and from African pig RVAs in particular makes it difficult to differentiate between the porcine or human origin of certain gene segments.

The child from which strain KisB332 was isolated lived in close proximity of a pig farm, providing a plausible route of transmission. Unfortunately it was not possible to take samples from the pigs living in close contact with the child. These findings suggest that KisB332 is of porcine origin and that this strain, after an interspecies transmission and potential reassortment with human RVAs, was able to cause gastro-enteritis in a human child. Surprisingly, a second G4P[6] strain (KisB603) was found in this study with very similar VP4 and VP7 gene sequences as those of KisB332. In theory, both strains can be the result of independent interspecies transmission events but a more plausible theory would be that the G4P[6] strain was able to spread from one human to another, although this hypothesis needs further investigation.

Although interspecies transmission events mostly result in so-called death-end infections, interspecies transmissions from pigs to humans seem to occur rather frequently, probably due to the fact that porcine RVA strains share a similar genetic background with human Wa-like RVAs [62]. Overall, these results underline the importance of complete genetic characterization of RVA strains and indicate that reassortments and interspecies transmission between human RVAs and porcine RVA strains occur frequently in the COD. Interspecies transmission events can have a potential negative influence on the efficacy of RVA vaccines, highlighting the need for continued surveillance of RVA diversity in the COD.

Based on the data from this study, RVA vaccines will be challenged with a wider variety of different G- and P- genotypes in the COD compared to those found in high-income countries. Of particular importance for the potential implementation of the currently available RVA vaccines in the COD is the predominance of P[6] detected in this study (52%) and the high proportion of strains with the G8 VP7 specificity (27%). Since these genotypes are not included in the currently available RVA vaccines and the genotype specific vaccine efficacy against these genotypes is not yet known, the success of vaccination programs could be lower. Nevertheless, implementation of rotavirus vaccines in the COD would be highly recommended to prevent rotavirus related morbidity and mortality.

## Supporting Information

Figure S1 **Phylogenetic dendrogram based on 595 nucleotides of the VP4 encoding gene of the 12 representative RVA strains characterized in the study (black triangle) and RVA strains isolated all over the world.** Tree was rooted using RVA/Human-tc/JPN/AU-1/1982/G3P9] as an outgroup. Bootstrap values (500 replicates) above 70 are shown.
(TIF)

Figure S2 **Phylogenetic dendrogram based on 503 nucleotides of the VP7 encoding gene of the 12 representative RVA strains characterized in this study (black triangle) and RVA strains isolated all over the world.** Tree was rooted using RVA/Pigeon-tc/JPN/PO-13/1983/G18P[17] as an outgroup. Bootstrap values (500 replicates) above 70 are shown.
(TIF)

Table S1 **Primers used to amplify the VP1, VP2, VP3, VP4, VP6, VP7, NSP1, NSP2, NSP3, NSP4 and NSP5 gene segments described in this study.**
(DOC)

## References

[1] Tate JE, Burton AH, Boschi-Pinto C, Steele AD, Duque J, et al (2012) the WHO-coordinated Global Rotavirus Surveillance Network. 2008 estimate of worldwide rotavirus-associated mortality in children younger than 5 years before the introduction of universal rotavirus vaccination programmes: a systematic review and meta-analysis. Lancet Infect Dis 12: 136–141. 22030330 10.1016/S1473-3099(11)70253-5

[2] Seheri M, Nemarude L, Peenze I, Netshifhefhe L, Nyaga MM, et al (2014) Update of rotavirus strains circulating in Africa from 2007 through 2011. Pediatr Infect Dis J 33 Suppl 1S76–84. 24343619 10.1097/INF.0000000000000053

[3] Jere KC, Mlera L, O’Neill HG, Peenze I, van Dijk AA (2012) Whole genome sequence analyses of three African bovine rotaviruses reveal that they emerged through multiple reassortment events between rotaviruses from different mammalian species. Vet Microbiol 159: 245–250. 22541163 10.1016/j.vetmic.2012.03.040

[4] Alkan F, Ozkul A, Oguzoglu TC, Timurkan MO, Caliskan E, et al (2010) Distribution of G (VP7) and P (VP4) genotypes of group A bovine rotaviruses from Turkish calves with diarrhea, 1997–2008. Vet Microbiol 141: 231–237. 19854003 10.1016/j.vetmic.2009.09.016

[5] Midgley SE, Banyai K, Buesa J, Halaihel N, Hjulsager CK, et al (2012) Diversity and zoonotic potential of rotaviruses in swine and cattle across Europe. Vet Microbiol 156: 238–245. 22079216 10.1016/j.vetmic.2011.10.027

[6] Matthijnssens J, Potgieter CA, Ciarlet M, Parreno V, Martella V, et al (2009) Are human P[14] rotavirus strains the result of interspecies transmissions from sheep or other ungulates that belong to the mammalian order Artiodactyla? J Virol 83: 2917–2929. 19153225 10.1128/JVI.02246-08 PMC2655590

[7] Monini M, Cappuccini F, Battista P, Falcone E, Lavazza A, et al (2008) Molecular characterization of bovine rotavirus strains circulating in northern Italy, 2003–2005. Vet Microbiol 129: 384–389. 18191347 10.1016/j.vetmic.2007.11.036

[8] Cunliffe NA, Gentsch JR, Kirkwood CD, Gondwe JS, Dove W, et al (2000) Molecular and serologic characterization of novel serotype G8 human rotavirus strains detected in Blantyre, Malawi. Virology 274: 309–320. 10964774 10.1006/viro.2000.0456

[9] Papp H, Laszlo B, Jakab F, Ganesh B, De Grazia S, et al (2013) Review of group A rotavirus strains reported in swine and cattle. Vet Microbiol 165: 190–199. 23642647 10.1016/j.vetmic.2013.03.020 PMC7117210

[10] Martella V, Banyai K, Ciarlet M, Iturriza-Gomara M, Lorusso E, et al (2006) Relationships among porcine and human P[6] rotaviruses: evidence that the different human P[6] lineages have originated from multiple interspecies transmission events. Virology 344: 509–519. 16194556 10.1016/j.virol.2005.08.029

[11] Nakagomi T, Doan YH, Dove W, Ngwira B, Iturriza-Gomara M, et al (2013) G8 rotaviruses with conserved genotype constellations detected in Malawi over 10 years (1997–2007) display frequent gene reassortment among strains co-circulating in humans. J Gen Virol 94: 1273–1295. 23407423 10.1099/vir.0.050625-0 PMC3945219

[12] Esona MD, Geyer A, Page N, Trabelsi A, Fodha I, et al (2009) Genomic characterization of human rotavirus G8 strains from the African rotavirus network: relationship to animal rotaviruses. J Med Virol 81: 937–951. 19319943 10.1002/jmv.21468

[13] Matthijnssens J, Rahman M, Yang X, Delbeke T, Arijs I, et al (2006) G8 rotavirus strains isolated in the Democratic Republic of Congo belong to the DS-1-like genogroup. J Clin Microbiol 44: 1801–1809. 16672410 10.1128/JCM.44.5.1801-1809.2006 PMC1479174

[14] Kabue JP, Peenze I, de Beer M, Esona MD, Lunfungula C, et al (2010) Characterization of human rotavirus recovered from children with acute diarrhea in Kinshasa, Democratic Republic Of Congo. J Infect Dis 202 Suppl: S193–197 10.1086/653576 20684702

[15] Nielsen NM, Eugen-Olsen J, Aaby P, Molbak K, Rodrigues A, et al (2005) Characterisation of rotavirus strains among hospitalised and non-hospitalised children in Guinea-Bissau, 2002 A high frequency of mixed infections with serotype G8. J Clin Virol 34: 13–21. 16087119 10.1016/j.jcv.2004.12.017

[16] Nokes DJ, Peenze I, Netshifhefhe L, Abwao J, De Beer MC, et al (2010) Rotavirus genetic diversity, disease association, and temporal change in hospitalized rural Kenyan children. J Infect Dis 202 Suppl: S180–186 10.1086/653566 PMC2923076 20684700

[17] Cunliffe NA, Ngwira BM, Dove W, Thindwa BD, Turner AM, et al (2010) Epidemiology of rotavirus infection in children in Blantyre, Malawi, 1997–2007. J Infect Dis 202 Suppl: S168–174 10.1086/653577 20684698

[18] Aminu M, Page NA, Ahmad AA, Umoh JU, Dewar J, et al (2010) Diversity of rotavirus VP7 and VP4 genotypes in Northwestern Nigeria. J Infect Dis 202 Suppl: S198–204 10.1086/653570 20684703

[19] Jere KC, Sawyerr T, Seheri LM, Peenze I, Page NA, et al (2011) A first report on the characterization of rotavirus strains in Sierra Leone. J Med Virol 83: 540–550. 21264877 10.1002/jmv.21999

[20] Binka FN, Anto FK, Oduro AR, Awini EA, Nazzar AK, et al (2003) Incidence and risk factors of paediatric rotavirus diarrhoea in northern Ghana. Trop Med Int Health 8: 840–846. 12950670 10.1046/j.1365-3156.2003.01097.x

[21] Yassin MA, Kirby A, Mengistu AA, Arbide I, Dove W, et al (2012) Unusual norovirus and rotavirus genotypes in Ethiopia. Paediatr Int Child Health 32: 51–55. 22525449 10.1179/1465328111Y.0000000047

[22] Ndze VN, Papp H, Achidi EA, Gonsu KH, Laszlo B, et al (2013) One year survey of human rotavirus strains suggests the emergence of genotype G12 in Cameroon. J Med Virol 85: 1485–1490. 23765785 10.1002/jmv.23603 PMC8167840

[23] Nordgren J, Bonkoungou IJ, Nitiema LW, Sharma S, Ouermi D, et al (2012) Rotavirus in diarrheal children in rural Burkina Faso: high prevalence of genotype G6P[6]. Infect Genet Evol 12: 1892–1898. 22964045 10.1016/j.meegid.2012.08.014

[24] Ruiz-Palacios GM, Perez-Schael I, Velazquez FR, Abate H, Breuer T, et al (2006) Safety and efficacy of an attenuated vaccine against severe rotavirus gastroenteritis. N Engl J Med 354: 11–22. 16394298 10.1056/NEJMoa052434

[25] Vesikari T, Matson DO, Dennehy P, Van Damme P, Santosham M, et al (2006) Safety and efficacy of a pentavalent human-bovine (WC3) reassortant rotavirus vaccine. N Engl J Med 354: 23–33. 16394299 10.1056/NEJMoa052664

[26] Armah GE, Sow SO, Breiman RF, Dallas MJ, Tapia MD, et al (2010) Efficacy of pentavalent rotavirus vaccine against severe rotavirus gastroenteritis in infants in developing countries in sub-Saharan Africa: a randomised, double-blind, placebo-controlled trial. Lancet 376: 606–614. 20692030 10.1016/S0140-6736(10)60889-6

[27] Madhi SA, Cunliffe NA, Steele D, Witte D, Kirsten M, et al (2010) Effect of human rotavirus vaccine on severe diarrhea in African infants. N Engl J Med 362: 289–298. 20107214 10.1056/NEJMoa0904797

[28] Sow SO, Tapia M, Haidara FC, Ciarlet M, Diallo F, et al (2012) Efficacy of the oral pentavalent rotavirus vaccine in Mali. Vaccine 30 Suppl 1A71–78. 22520140 10.1016/j.vaccine.2011.11.094

[29] Johns Hopkins Bloomberg School of Public Health International Vaccine Access Center. (2014) VIMS Report: Global Vaccine Introduction. Database: VIMS http://www.jhsph.edu/research/centers-and-institutes/ivac/vims/IVAC-VIMS-Report-2014-Mar.pdf. Accessed 4 June 2014.

[30] PATH (2014) Rotavirus vaccines access and delivery. Country introductions of rotavirus vaccines. Available: http://sites.path.org/rotavirusvaccine/rotavirus-advocacy-and-communications-toolkit/country-introduction-maps-and-list/. Accessed 4 June 2014.

[31] Moon SS, Tate JE, Ray P, Dennehy PH, Archary D, et al.. (2013) Differential Profiles and Inhibitory Effect on Rotavirus Vaccines of Non-Antibody Components in Breast Milk from Mothers in Developing and Developed Countries. Pediatr Infect Dis J. 10.1097/INF.0b013e318290646d PMC4610365 23584581

[32] Serazin AC, Shackelton LA, Wilson C, Bhan MK (2010) Improving the performance of enteric vaccines in the developing world. Nat Immunol 11: 769–773. 20720580 10.1038/ni0910-769

[33] Hu L, Crawford SE, Czako R, Cortes-Penfield NW, Smith DF, et al (2012) Cell attachment protein VP8* of a human rotavirus specifically interacts with A-type histo-blood group antigen. Nature 485: 256–259. 22504179 10.1038/nature10996 PMC3350622

[34] Nyaga MM, Jere KC, Peenze I, Mlera L, van Dijk AA, et al (2013) Sequence analysis of the whole genomes of five African human G9 rotavirus strains. Infect Genet Evol 16: 62–77. 23369762 10.1016/j.meegid.2013.01.005

[35] Jere KC, Mlera L, O’Neill HG, Potgieter AC, Page NA, et al (2011) Whole genome analyses of African G2, G8, G9, and G12 rotavirus strains using sequence-independent amplification and 454(R) pyrosequencing. J Med Virol 83: 2018–2042. 21915879 10.1002/jmv.22207

[36] Esona MD, Mijatovic-Rustempasic S, Foytich K, Roy S, Banyai K, et al (2013) Human G9P[8] rotavirus strains circulating in Cameroon, 1999–2000: Genetic relationships with other G9 strains and detection of a new G9 subtype. Infect Genet Evol 18C: 315–324. 10.1016/j.meegid.2013.06.005 PMC4604607 23770141

[37] Ghosh S, Gatheru Z, Nyangao J, Adachi N, Urushibara N, et al (2011) Full genomic analysis of a G8P[1] rotavirus strain isolated from an asymptomatic infant in Kenya provides evidence for an artiodactyl-to-human interspecies transmission event. J Med Virol 83: 367–376. 21181935 10.1002/jmv.21974

[38] Jere KC, Mlera L, Page NA, van Dijk AA, O’Neill HG (2011) Whole genome analysis of multiple rotavirus strains from a single stool specimen using sequence-independent amplification and 454(R) pyrosequencing reveals evidence of intergenotype genome segment recombination. Infect Genet Evol 11: 2072–2082. 22019521 10.1016/j.meegid.2011.09.023

[39] Rahman M, Goegebuer T, De Leener K, Maes P, Matthijnssens J, et al (2004) Chromatography paper strip method for collection, transportation, and storage of rotavirus RNA in stool samples. J Clin Microbiol 42: 1605–1608. 15071012 10.1128/JCM.42.4.1605-1608.2004 PMC387597

[40] Matthijnssens J, Rahman M, Martella V, Xuelei Y, De Vos S, et al (2006) Full genomic analysis of human rotavirus strain B4106 and lapine rotavirus strain 30/96 provides evidence for interspecies transmission. J Virol 80: 3801–3810. 16571797 10.1128/JVI.80.8.3801-3810.2006 PMC1440464

[41] Tamura K, Peterson D, Peterson N, Stecher G, Nei M, et al (2011) MEGA5: molecular evolutionary genetics analysis using maximum likelihood, evolutionary distance, and maximum parsimony methods. Mol Biol Evol 28: 2731–2739. 21546353 10.1093/molbev/msr121 PMC3203626

[42] Gomez MM, da Silva MF, Zeller M, Heylen E, Matthijnssens J, et al (2013) Phylogenetic analysis of G1P[6] group A rotavirus strains detected in Northeast Brazilian children fully vaccinated with Rotarix. Infect Genet Evol 19: 395–402. 23538335 10.1016/j.meegid.2013.03.028

[43] Papp H, Borzak R, Farkas S, Kisfali P, Lengyel G, et al (2013) Zoonotic transmission of reassortant porcine G4P[6] rotaviruses in Hungarian pediatric patients identified sporadically over a 15 year period. Infect Genet Evol 19: 71–80. 23792183 10.1016/j.meegid.2013.06.013

[44] Kim HH, Matthijnssens J, Kim HJ, Kwon HJ, Park JG, et al (2012) Full-length genomic analysis of porcine G9P[23] and G9P[7] rotavirus strains isolated from pigs with diarrhea in South Korea. Infect Genet Evol 12: 1427–1435. 22613801 10.1016/j.meegid.2012.04.028

[45] Okitsu S, Khamrin P, Thongprachum A, Kongkaew A, Maneekarn N, et al (2013) Whole-genomic analysis of G3P[23], G9P[23] and G3P[13] rotavirus strains isolated from piglets with diarrhea in Thailand, 2006–2008. Infect Genet Evol 18: 74–86. 23681022 10.1016/j.meegid.2013.05.005

[46] Martel-Paradis O, Laurin MA, Martella V, Sohal JS, L’Homme Y (2013) Full-length genome analysis of G2, G9 and G11 porcine group A rotaviruses. Vet Microbiol 162: 94–102. 23017831 10.1016/j.vetmic.2012.08.028

[47] Degiuseppe JI, Beltramino JC, Millan A, Stupka JA, Parra GI (2013) Complete genome analyses of G4P[6] rotavirus detected in Argentinean children with diarrhoea provides evidence of interspecies transmission from swine. Clin Microbiol Infect 19: E367–371. 23586655 10.1111/1469-0691.12216

[48] Mukherjee A, Ghosh S, Bagchi P, Dutta D, Chattopadhyay S, et al (2011) Full genomic analyses of human rotavirus G4P[4], G4P[6], G9P[19] and G10P[6] strains from North-eastern India: evidence for interspecies transmission and complex reassortment events. Clin Microbiol Infect 17: 1343–1346. 21884295 10.1111/j.1469-0691.2010.03383.x

[49] Dong HJ, Qian Y, Huang T, Zhu RN, Zhao LQ, et al (2013) Identification of circulating porcine-human reassortant G4P[6] rotavirus from children with acute diarrhea in China by whole genome analyses. Infect Genet Evol 20: 155–162. 24012957 10.1016/j.meegid.2013.08.024

[50] Wang YH, Kobayashi N, Nagashima S, Zhou X, Ghosh S, et al (2010) Full genomic analysis of a porcine-bovine reassortant G4P[6] rotavirus strain R479 isolated from an infant in China. J Med Virol 82: 1094–1102. 20419827 10.1002/jmv.21760

[51] Maestri RP, Kaiano JH, Neri DL, Soares Lda S, Guerra Sde F, et al (2012) Phylogenetic analysis of probable non-human genes of group A rotaviruses isolated from children with acute gastroenteritis in Belem, Brazil. J Med Virol 84: 1993–2002. 23080508 10.1002/jmv.23364

[52] Heylen E, Zeller M, Ciarlet M, De Coster S, Van Ranst M, et al (2013) Complete genetic characterization of human G2P[6] and G3P[6] rotavirus strains. Infect Genet Evol 13: 27–35. 22982160 10.1016/j.meegid.2012.08.019

[53] Rahman M, De Leener K, Goegebuer T, Wollants E, Van der Donck I, et al (2003) Genetic characterization of a novel, naturally occurring recombinant human G6P[6] rotavirus. J Clin Microbiol 41: 2088–2095. 12734253 10.1128/JCM.41.5.2088-2095.2003 PMC154672

[54] Matthijnssens J, Rahman M, Van Ranst M (2008) Two out of the 11 genes of an unusual human G6P[6] rotavirus isolate are of bovine origin. J Gen Virol 89: 2630–2635. 18796733 10.1099/vir.0.2008/003780-0

[55] Banyai K, Esona MD, Kerin TK, Hull JJ, Mijatovic S, et al (2009) Molecular characterization of a rare, human-porcine reassortant rotavirus strain, G11P[6], from Ecuador. Arch Virol 154: 1823–1829. 19763776 10.1007/s00705-009-0499-1

[56] Bucardo F, Rippinger CM, Svensson L, Patton JT (2012) Vaccine-derived NSP2 segment in rotaviruses from vaccinated children with gastroenteritis in Nicaragua. Infect Genet Evol 12: 1282–1294. 22487061 10.1016/j.meegid.2012.03.007 PMC3372771

[57] Clark HF, Hoshino Y, Bell LM, Groff J, Hess G, et al (1987) Rotavirus isolate WI61 representing a presumptive new human serotype. J Clin Microbiol 25: 1757–1762. 2443534 10.1128/jcm.25.9.1757-1762.1987 PMC269322

[58] Zeller M, Heylen E, De Coster S, Van Ranst M, Matthijnssens J (2012) Full genome characterization of a porcine-like human G9P[6] rotavirus strain isolated from an infant in Belgium. Infect Genet Evol 12: 1492–1500. 22430049 10.1016/j.meegid.2012.03.002

[59] Bridger JC, Woode GN (1975) Neonatal calf diarrhoea: identification of a reovirus-like (rotavirus) agent in faeces by immunofluorescence and immune electron microscopy. Br Vet J 131: 528–535. 172192

[60] Cook SM, Glass RI, LeBaron CW, Ho MS (1990) Global seasonality of rotavirus infections. Bull World Health Organ 68: 171–177. 1694734 PMC2393128

[61] Levy K, Hubbard AE, Eisenberg JN (2009) Seasonality of rotavirus disease in the tropics: a systematic review and meta-analysis. Int J Epidemiol 38: 1487–1496. 19056806 10.1093/ije/dyn260 PMC2800782

[62] Matthijnssens J, Ciarlet M, Heiman E, Arijs I, Delbeke T, et al (2008) Full genome-based classification of rotaviruses reveals a common origin between human Wa-Like and porcine rotavirus strains and human DS-1-like and bovine rotavirus strains. J Virol 82: 3204–3219. 18216098 10.1128/JVI.02257-07 PMC2268446

