# Supplementary figures and images for: Rotavirus Surveillance in Kisangani, the Democratic Republic of the Congo, Reveals a High Number of Unusual Genotypes and Gene Segments of Animal Origin in Non-Vaccinated Symptomatic Children
Source: PLoS One. 2014 Jun 26;9(6):e100953. doi: 10.1371/journal.pone.0100953 (PMC4072759; doi:10.1371/journal.pone.0100953)

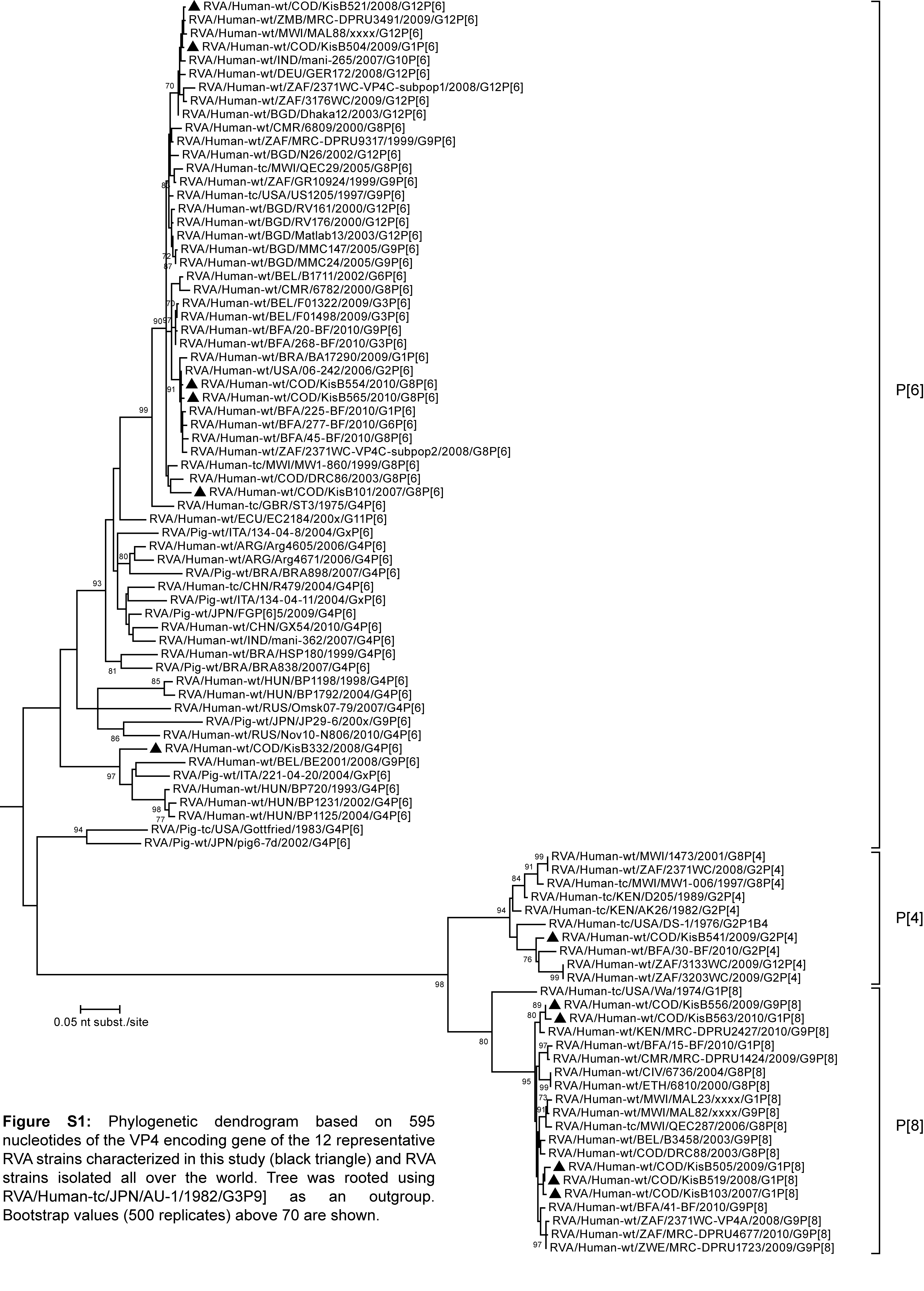

Supplement: Figure S1 — Phylogenetic dendrogram based on 595 nucleotides of the VP4 encoding gene of the 12 representative RVA strains characterized in the study (black triangle) and RVA strains isolated all over the world. Tree was rooted using RVA/Human-tc/JPN/AU-1/1982/G3P9] as an outgroup. Bootstrap values (500 replicates) above 70 are shown. (TIF) [file pone.0100953.s001.tif]

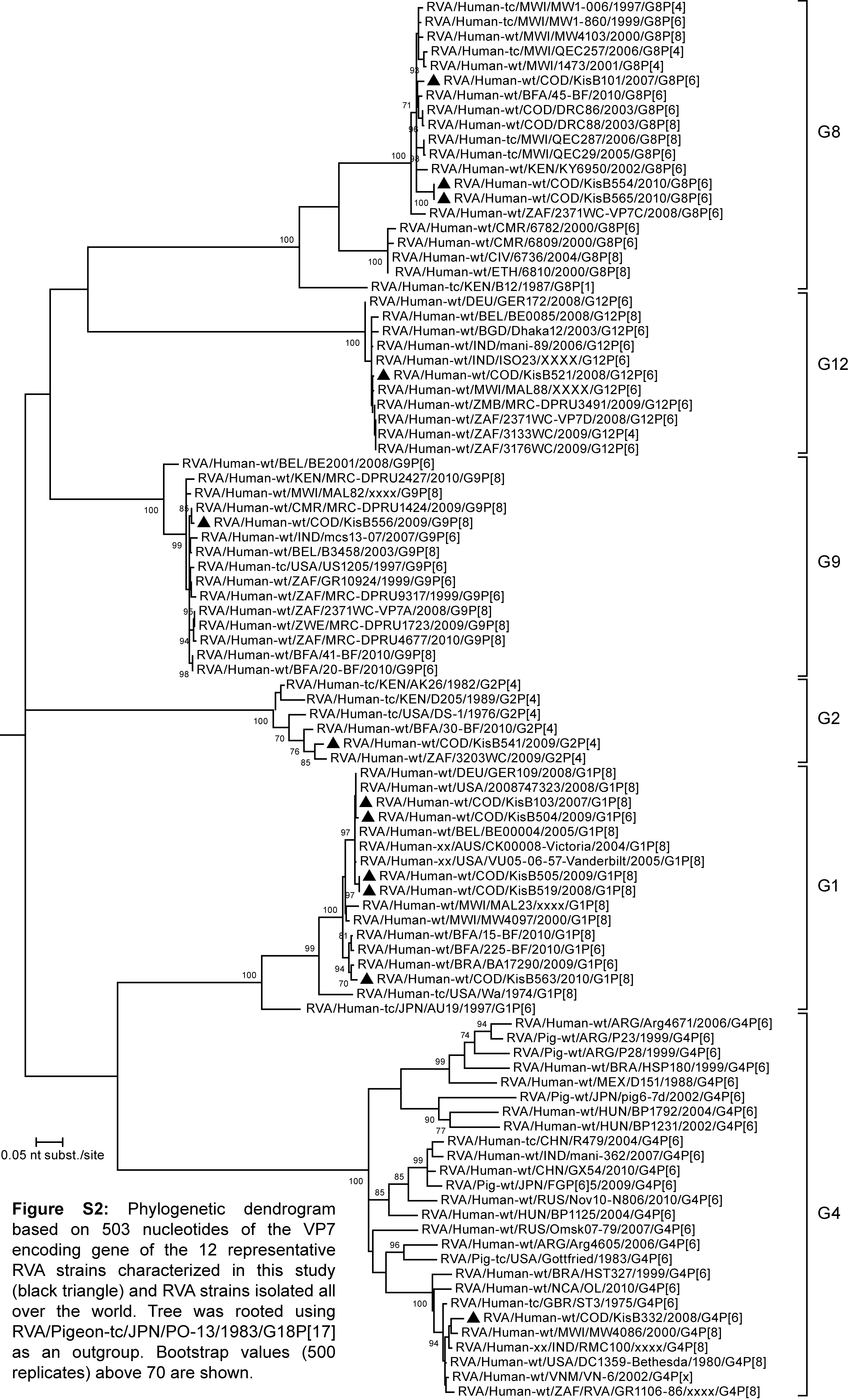

Supplement: Figure S2 — Phylogenetic dendrogram based on 503 nucleotides of the VP7 encoding gene of the 12 representative RVA strains characterized in this study (black triangle) and RVA strains isolated all over the world. Tree was rooted using RVA/Pigeon-tc/JPN/PO-13/1983/G18P[17] as an outgroup. Bootstrap values (500 replicates) above 70 are shown. (TIF) [file pone.0100953.s002.tif]
