# Supplementary material for: Rotavirus Surveillance in Kisangani, the Democratic Republic of the Congo, Reveals a High Number of Unusual Genotypes and Gene Segments of Animal Origin in Non-Vaccinated Symptomatic Children
Source: PLoS One. 2014 Jun 26;9(6):e100953. doi: 10.1371/journal.pone.0100953 (PMC4072759; doi:10.1371/journal.pone.0100953)
Supplement: Table S1 — Primers used to amplify the VP1, VP2, VP3, VP4, VP6, VP7, NSP1, NSP2, NSP3, NSP4 and NSP5 gene segments described in this study. (DOC) [file pone.0100953.s003.doc]

**Table S1.** Primers used to amplify the VP1, VP2, VP3, VP4, VP6, VP7, NSP1, NSP2, NSP3, NSP4 and NSP5 gene segments described in this study.

| Gene segment | Primer name | (Degenerate) primer sequences * |
| --- | --- | --- |
| VP1 | GEN_VP1Fb ^¶^ | 5’-GGC TAT TAA AGC T**R**T ACA ATG GGG AAG-3' |
|  | GEN_VP1Rb ^¶^ | 5’-GGT CAC ATC TAA GCG **Y**TC TAA TCT TG -3' |
| VP2 | GEN_VP2Fc ^¶^ | 5’-GGC TAT TAA AGG **Y**TC AAT GGC GTA CAG-3' |
|  | GEN_VP2_Rbc ^¶^ | 5’-GTC ATA TCT CCA CA**R** TGG GGT TGG -3' |
| VP3 | GEN_VP3Fe ^¶^ | 5’-GGC T**W**T TAA AGC A**R**T ATT AGT AGT G-3’ |
|  | GEN_VP3_2584R | 5'- TGA CYA GTG TGT TAA GTT TYT AGC -3' |
| VP4 | VP4-1-17F | 5’-GGCTATAAAATGGCTTCGC-3’ |
|  | GEN_VP4_P6_2359R | 5'- GGT CAC ATC CTC TAT AGA GCT CTC -3' |
| VP6 | GEN_VP6F ^¶^ | 5’-GGC TTT **W**AA ACG AAG TCT TC -3 |
|  | GEN_VP6R | 5’-GGT CAC ATC CTC TCA CT -3’ |
| VP7 | BEG9 | 5’-GGCTTTAAAAGAGAGAATTTCCGTCTGG-3’ |
|  | END9 | 5’-GGTCACATCATACAATTCTAATCTAAG-3’ |
| NSP1 | GEN_NSP1F | 5’-GGC TTT TTT TTA TGA AAA GTC TTG -3’ |
|  | GEN_NSP1R | 5'-GGT CAC ATT TTA TGC TGC C -3’ |
| NSP2 | GEN_NSP2F | 5’- GGC TTT TAA AGC GTC TCA G -3’ |
|  | GEN_NSP2R | 5’- GGT CAC ATA AGC GCT TTC -3’ |
| NSP3 | GEN_NSP3F | 5’- GGC TTT TAA TGC TTT TCA GTG -3’ |
|  | GEN_NSP3R | 5’- ACA TAA CGC CCC TAT AGC -3’ |
| NSP4 | GEN_NSP4F | 5’- GGC TTT TAA AAG TTC TGT TCC -3’ |
|  | GEN_NSP4R ^¶^ | 5’- GG**W** **Y**AC **RY**T AAG ACC **R**TT CC -3’ |
| NSP5 | GEN_NSP5F | 5’-GGC TTT TAA AGC GCT ACA G -3’ |
|  | GEN_NSP5R | 5’-GGT CAC AAA ACG GGA GT  -3’ |

* All the primers are located at the 5’ and 3’ ends of the respective gene segments, except for primer GEN_VP2_Rbc, which starts at nucleotide 2 starting from the 3’. ¶: Degenerate primers; R = A/G, W = A/T, Y = C/T.
